# Protease activated receptor 1-induced glutamate release in cultured astrocytes is mediated by Bestrophin-1 channel but not by vesicular exocytosis

**DOI:** 10.1186/1756-6606-5-38

**Published:** 2012-10-12

**Authors:** Soo-Jin Oh, Kyung-Seok Han, Hyungju Park, Dong ho Woo, Hye Yun Kim, Stephen F Traynelis, C Justin Lee

**Affiliations:** 1 Center for Neural Science and Center for Functional Connectomics, Korea Institute of Science and Technology (KIST), Seoul, South Korea; 2 Neuroscience Program, University of Science and Technology (UST), Daejeon, South Korea; 3 Department of Pharmacology, School of Medicine, Emory University, Atlanta, GA, USA

**Keywords:** Astrocyte, Bestrophin-1, Glutamate

## Abstract

**Background:**

Glutamate is the major transmitter that mediates the principal form of excitatory synaptic transmission in the brain. It has been well established that glutamate is released via Ca^2+^-dependent exocytosis of glutamate-containing vesicles in neurons. However, whether astrocytes exocytose to release glutamate under physiological condition is still unclear.

**Findings:**

We report a novel form of glutamate release in astrocytes via the recently characterized Ca^2+^-activated anion channel, Bestrophin-1 (Best1) by Ca^2+^ dependent mechanism through the channel pore. We demonstrate that upon activation of protease activated receptor 1 (PAR1), an increase in intracellular Ca^2+^ concentration leads to an opening of Best1 channels and subsequent release of glutamate in cultured astrocytes.

**Conclusions:**

These results provide strong molecular evidence for potential astrocyte-neuron interaction via Best1-mediated glutamate release.

## Background

Astrocytes and neurons are intimate partners in the brain. Astrocytes respond to neuronal activity by an increase in intracellular Ca^2+^ ([Ca^2+^_i_) [1,2]. The synaptically released neurotransmitters such as glutamate and ATP activate corresponding G_αq_ protein-coupled receptors expressed in astrocytes, resulting in increase in [Ca^2+^_i_ and Ca^2+^-dependent release of gliotransmitters from astrocytes [2,3]. These gliotransmitters in turn influence the neuronal excitability and synaptic activities [3-8].

At the heart of this neuron-glia interaction there is glutamate, which is released from neurons by synaptic vesicles to mediate neuron-to-glia communication and from astrocytes by various Ca^2+^-dependent mechanisms to mediate glia-to-neuron communication [9,10]. Therefore, understanding how astrocytes release glutamate in Ca^2+^-dependent manner and how astrocytically-released glutamate regulates neuronal synaptic activity has been a major challenge. One line of accumulating evidence supports that astrocytes release glutamate by Ca^2+^- and SNARE-dependent exocytosis mechanism [11], although this mechanism has been recently challenged [12]. It has been reported that glutamate released by SNARE-dependent mechanism regulates hippocampal synapse between perforant path afferents and granule cells [13] or Schaffer Collateral afferent and CA1 pyramidal neurons [6] through N-methyl-D-aspartic acid receptor (NMDAR) activation. Yet, astrocytes also express a combination of anion-permeable channels to move Cl^-^ and large anions such as glutamate across membranes in response to specific physiological stimulations [14,15], raising the possibility that astrocytes have a multiple glutamate release mechanisms. Although astrocytes under physiological conditions appear to possess a Ca^2+^- and anion channel-dependent glutamate release mechanism [10,16], until now this idea has not been directly demonstrated due to the lack of molecular evidence.

*Bestrophin* is the gene identified as the gene responsible for Best's vitelliform macular dystrophy and has been shown to encode a functional Ca^2+^-activated anion channel (CAAC) in nonneuronal tissue and peripheral neurons [17]. This Bestrophin-1 channel (Best1) is directly activated by submicromolar intracellular Ca^2+^ concentration and has an anion selective pore with single channel activities [17-24]. Recently, we have discovered that astrocytes express CAAC and that *Best1* encodes most of the CAAC in astrocytes [25]. This astrocytic CAAC showed a considerable permeability to large anions such as isethionate and glutamate [25]. In addition, we have recently demonstrated that Best1 channel mediates tonic GABA release from cerebellar glia by a direct permeation [26]. Therefore, we hypothesized that Best1 is the molecular identity of Ca^2+^-dependent anion channel that mediates glutamate release from astrocytes. Here we tested whether Best1 is an alternative Ca^2+^-dependent glutamate release mechanism in cultured astrocytes. To stimulate astrocytes more physiologically, we activated endogenous GPCRs by application of TFLLR [27] a selective peptide agonist of the protease activated receptor 1 (PAR1). We demonstrate that PAR1-induced glutamate release is mediated not by conventional vesicular exocytosis but by an activation of glutamate permeable anion channel Best1 in cultured astrocytes.

## Results

### PAR1-induced glutamate release is mediated by CAAC

**Figure 1:**
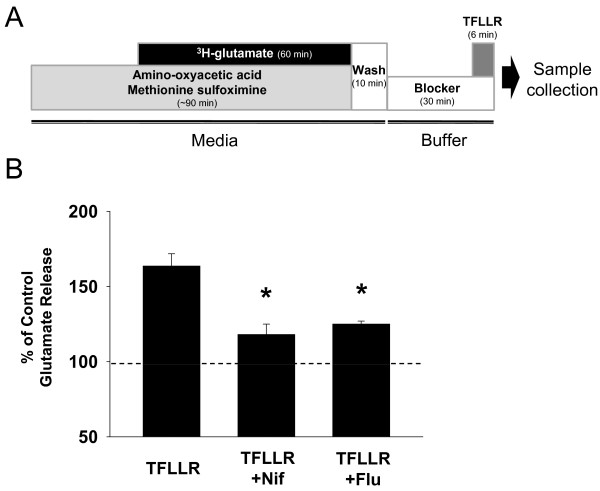
**PAR1 induced glutamate release is blocked by CAAC blockers. A**) The experimental scheme of *in vitro* glutamate release assay using isotope-labeled glutamate (^3^H-glutamate). 30 μM of TFLLR was treated for 6 min to induce glutamate release from cultured astrocytes. **B**) Bar graphs represent the averaged relative amount of released glutamate by TFLLR treatment (n=12). 100 μM of Niflumic acid (Nif, n=11) or Flufenamic acid (Flu, n=4) was pretreated to block anion channel activation. **p < 0.05 vs. TFLLR-treated group.

It has been suggested that hippocampal astrocytes utilize an anion channel-dependent mechanism for Ca^2+^-induced glutamate release [10,16]. To confirm the anion channel-mediated glutamate release from astrocytes, we performed an *in vitro* glutamate release assay using radioactive isotope-labeled glutamate (^3^H-glutamate) in cultured hippocampal astrocytes (Figure 1A) [7]. To elicit intracellular Ca^2+^ increase and the subsequent release of glutamate in astrocytes, we used TFLLR to activate PAR1. We found that an application of TFLLR induced a significant glutamate release from cultured astrocytes (Figure 1B), which was inhibited by a pre-incubation of anion channel blockers, such as niflumic acid and flufenamic acid (Figure 1B) [28]. These results suggested that astrocytes utilize anion channel-dependent glutamate release mechanism.

### Best1 mediates a Ca^2+^-dependent glutamate release in cultured astrocytes

**Figure 2:**
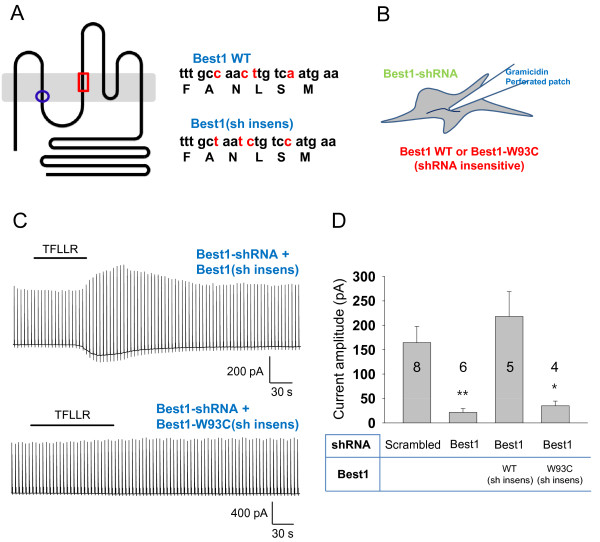
**Development of Best1 specific genetic tools. A**) Left; Topology model of Bestrophin suggested by Tsunenari et al [17]. Blue circle indicates amino acid position of Best1 pore mutant (W93C). Red square indicates target amino acids for Best1 shRNA. Right; nucleotide and amino acid sequences of Best1 shRNA target. Nucleotide sequence differences from wild type Best1 (Best1 WT) and shRNA insensitive Best1 (Best1(sh insens)) were highlightened. **B**) Schematic diagram for perforated-patch-current recording from cultured astrocytes. **C**) Representative perforated-patch-current recording from Best1-shRNA and Best1 (sh insens) or Best1-shRNA and shRNA insensitive pore mutant Best1 (Best1-W93C (sh insens)) expressing cultured astrocytes. The current responses were recorded in response to a voltage ramp command (from -100 to +100 mV, 1 s duration, 0.2 Hz; V_h_ of -70 mV) before and after 30 μM TFLLR treatment. **D**) The bar graph summarizes the mean current amplitude at V_h_ = -70 mV ± s.e.m (*p < 0.05, **p < 0.01, unpaired t-test). Among 13 recorded astrocytes which expressed Best1-shRNA and Best1 (sh insens), only 5 cells showed comparable currents. And a big current recorded in an astrocyte expressing Best1-shRNA and Best1-W93C (sh insens) was eliminated in the bar graph. Numbers of determinations are indicated on the bar graph.

**Figure 3:**
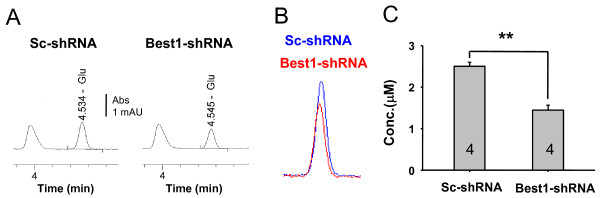
**HPLC detection of TFLLR-induced glutamate release from astrocytes. A**) The raw traces of HPLC detection of TFLLR-induced glutamate release from scrambled-shRNA (Sc-shRNA) or Best1-shRNA expressing cultured astrocytes. **B**) The merged traces are from Sc-shRNA (blue) and B1-shRNA (red) infected astrocytes. **C**) Bar graphs show the averaged HPLC-detected glutamate peak level. **p < 0.01.

We have recently demonstrated that Best1 channel is activated by intracellular Ca^2+^ (EC_50_ = 150 nM) with considerable permeability to GABA [26]. To test whether Ca^2+^- induced glutamate release is also mediated by Best1 in native astrocytes, we adopted a gene silencing tool using the short hairpin RNA (shRNA) specifically targeted to mouse Best1 transcript [25,26]. We recorded whole cell currents in cultured astrocytes under gramicidin-D perforated patch configuration (Figure 2B). We found the significant Ca^2+^-induced conductance carried by the efflux of glutamate at -70 mV in astrocyte with scrambled-shRNA. Silencing the Best1 gene via specific shRNA for Best1 significantly eliminated PAR1 induced whole-cell current in cultured astrocytes. This effect was fully rescued by the cotransfection of Best1-shRNA, along with a shRNA-insensitive form of Best1 (Figure 2C and D). However, the addition of a pore mutation at position 93, from tryptophan to cystein (Best1-W93C sh insens) [29] failed to rescue the current (Figure 2C and D). These results indicate that pore of Best1 channel is responsible for the permeation of anions such as Cl^-^ and glutamate. We further tested the effect of Best1-shRNA on glutamate release using conventional high-performance liquid chromatography (HPLC) from the solution samples collected after 5 min application of TFLLR in cultured astrocytes. In this experiment, we firstly prepared astrocytes with the same cell number of cells that were transfected with Sc-shRNA or Best1-shRNA. Since we found that Best1-shRNA did not affect astrocytic cell growth or survival (data not shown), we assumed that total number of astrocytes in each sample after transfection of Sc-shRNA or Best1-shRNA should be similar. We found a decrease in glutamate release by Best1-shRNA using HPLC method (Figure 3). These results indicate that the Best1 channel is the molecular identity of glutamate permeable CAAC and suggest that the Best1 channel might be the molecular machinery for Ca^2+^-dependent glutamate release from astrocytes.

**Figure 4:**
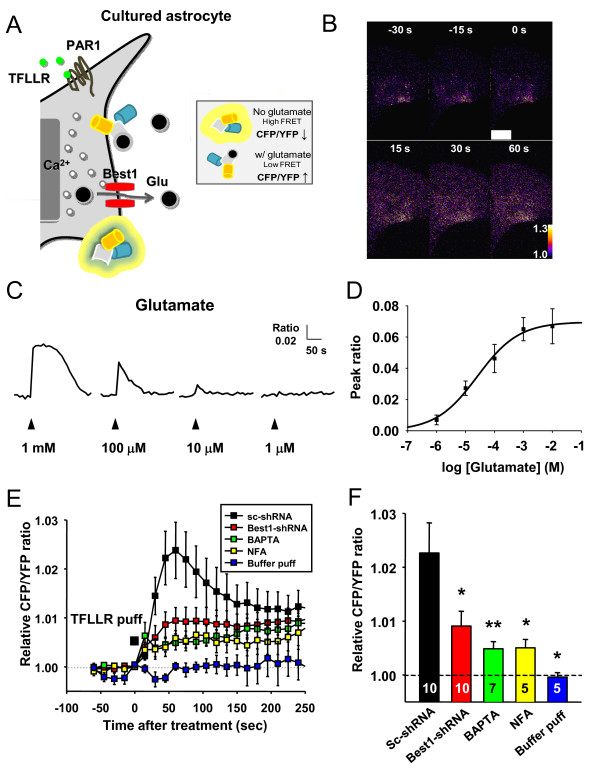
**Best1-mediated glutamate release from cultured astrocytes by using glutamate-sensitive FRET sensor, GluSFnR. A**) Schematic diagram of principles of FRET-based glutamate sensor and imaging. Released glutamate from astrocyte binds to glutamate binding motif of GluSnFR (glutamate FRET sensor), resulting in decrease of FRET between CFP and YFP or increased CFP/YFP ratio. **B**) Representative FRET images from GluSnFR-expressing cultured astrocytes processed by CFP/YFP emission ratio. The white bar shown at 0 s image indicates the puff of TFLLR (500 μM, 100 msec). **C**) Astrocytic GluSFnR responses (CFP/YFP ratio change) by respective puff-treated extracellular glutamate concentration at the time point indicated by an arrow. **D**) The graph showing the relationship between glutamate concentration and peak value of relative CFP/YFP ratio. The calculated EC_50_ was ~ 25.57 μM. **E**) The graph shows the averaged relative CFP/YFP ratio values from time-lapse imaging using GluSFnR expressing-cultured astrocytes. Scrambled shRNA-expressing astrocytes (Sc-shRNA); Best1-shRNA-expressing astrocytes (B1-shRNA); naïve astrocytes pretreated with BAPTA-AM (BAPTA; 30 μM); naïve astrocytes pretreated with niflumic acid (NFA; 100 μM); naïve astrocytes treated with recording buffer puff (Buffer puff). **F**) Bar graph representing averaged relative CFP/YFP ratio (analyzed during the period indicated by the gray box in Figure 1F). *p < 0.05, **p < 0.01 vs. Sc-shRNA-expressing group. Numbers on each bar indicate number of cells from at least three independent culture batches.

To independently confirm the glutamate release by HPLC method, we tested the effect of Best1-shRNA on glutamate release using fluorescence resonance energy transfer (FRET) based glutamate sensor. The released glutamate was monitored using a glutamate-sensing fluorescent reporter, GluSnFR, which senses glutamate by changing the ratio of FRET from CFP to YFP upon glutamate binding [30] (Figure 4A). The concentration-response curve for glutamate on the sensor was within the range of estimated glutamate release from a single astrocyte in culture (~10^-6^ M) [7] (Figure 4C and 4D). The calculated EC_50_ of glutamate was ~25.57 μM (Figure 4D). We observed that a brief pressure application of TFLLR caused a long lasting glutamate release as indicated by the relative change in FRET ratio (Figure 4B and 4E). The amount of glutamate release was on average significantly lower in the astrocytes expressing Best1-shRNA (Figure 4E, red symbols) than those expressing control scrambled shRNA (Figure 4E, black symbols). We found a similar degree of decrease in glutamate release by Best1-shRNA using the FRET based glutamate imaging, as compared to HPLC method (Figures 3C and 4F). Co-application of TFLLR and niflumic acid or treating the cells with BAPTA-AM, significantly reduced the glutamate release from astrocytes (Figure 4E and 4F).

### PAR1 induced glutamate release is not due to vesicular release

**Figure 5:**
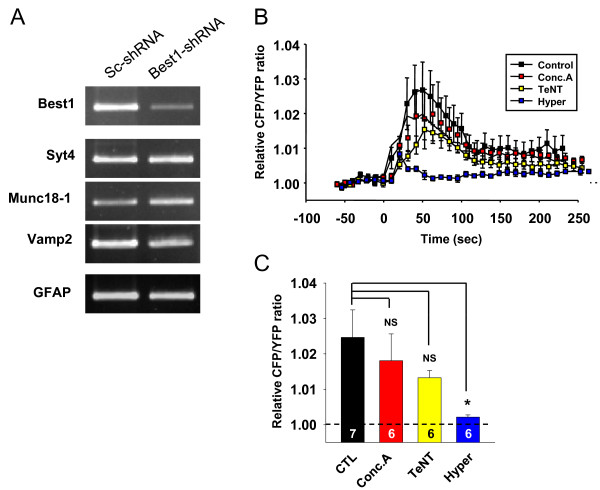
**TFLLR-induced glutamate is not by vesicular exocytosis. A**) To test whether Best1–shRNA affects the expression of exocytotic protein transcripts, mRNA expression levels of several endogenous vesicular machineries such as Syt4, Munc18-1, Vamp2 were analyzed from cultured astrocytes expressing Best1–shRNA or scrambled shRNA at least for 72 h. PCR cycles for Best1, Syt4 (Synaptotagmin4), Munc18-1, Vamp2 (Vesicle-associated membrane protein 2), and GFAP were 35, 35, 30, 30, and 25, respectively. **B**) Time-lapse imaging of relative CFP/YFP ratio from GluSFnR-expressing cultured astrcytes plotted as mean ± sem. Arrowhead indicates the time point of 500 μM TFLLR puff. Naïve astrocytes (Control); naïve astrocytes pretreated with 0.5 mM concanamycin (Conc.A; 2 hrs); naïve astrocytes pretreated with 10 nM tetanus toxin (TeNT; ~14 hrs); naïve astrocytes treated with hyperosmotic solution puff (Hyper). Bar graphs represent the averaged relative CFP/YFP ratio from 30 sec to 70 sec. Numbers of cells from at least two independent culture batches are indicated on the bar graph.

We noted that the amount of released glutamate was not completely inhibited by gene-silencing of Best1 (Figure 4E and 4F), raising a possibility that astrocytes have other Best1-independent mechanisms for glutamate release such as vesicular exocytosis. It is possible that Best1-shRNA affects the expression level of other genes involved in vesicular release. To address this possibility, we performed semi-quantitative RT-PCR analysis of cultured astrocytes with Best1-shRNA. Best1-shRNA showed a knock down efficiency for Best1 of over 90% while not affecting the expression level of other known genes such as Syt4 (Synaptotagmin4), Munc18-1, Vamp2 (Vesicle-associated membrane protein 2) involved in vesicular release (Figure 5A). We also tested whether inhibiting vesicular release machinery affects the glutamate release by using FRET based glutamate imaging. We found that the pretreatment with Conconomycin A (preventing vesicular glutamate release by inhibiting vesicular hydrogen ATPase, [31]) and Tetanus toxin (interfering all vesicular releases by inhibiting fusion of vesicles, [32]) slightly but not significantly reduced TFLLR-induced glutamate release from cultured astrocytes (Figure 5B and 5C). Consistent with these results, hyperosmotic challenge, which is known to cause a Ca^2+^ independent exocytosis of glutamate containing releasable vesicles, did not show any significant increase in glutamate release (Figure 5B and 5C). Taken together, PAR1-induced glutamate release is not mediated by vesicular exocytosis in cultured astrocytes.

## Discussion

In this study we report the direct evidence of anion channel-mediated glutamate release mechanism. We provide a series of evidence for Ca^2+^-activated, Best1-mediated glutamate release from astrocytes. For example, the selective gene silencing of astrocytic Best1 channels significantly reduced GPCR-induced and Ca^2+^-dependent glutamate release from astrocytes as measured by HPLC detection of glutamate from cultured astrocytes (Figure 3), and by FRET glutamate sensor in cultured astrocytes (Figure 4). The FRET based glutamate sensor has been well-characterized in the previous report [30]. In that report it has been demonstrated that FRET change is quite specific for glutamate because the sensor shows binding kinetics and high selectivity toward glutamate with no apparent change in ratio with APV, NBQX, NMDA, KA, AMPA, etc. [30]. We performed a calibration experiment by measuring glutamate EC_50_ in our imaging system and experimental conditions. Our calibration results showed that the FRET-based glutamate sensor in cultured astrocyte can be useful in detecting micromolar and submicromolar concentrations of released extracellular glutamate from a single astrocyte.

Until now, it has been proposed that glutamate could be released from astrocytes through multiple routes, including Ca^2+^- and SNARE-dependent vesicular exocytosis, the reversal action of glutamate transporters, transportation by cystine-glutamate antiporter, and permeation through channels or receptors, such as P2X7 receptor, volume regulated anion channel (VRAC), or gap junction hemichannel [33]. Among these, the molecular mechanism of Ca^2+^-dependent glutamate release has been extensively studied mainly because the generation of astrocytic Ca^2+^ transient responding to neuronal activity is one of the most important physiological readout of neural activity [3,5,7,10]. Numerous studies have proposed the SNARE-dependent exocytosis of glutamate as a potential route for astrocytic Ca^2+^-dependent glutamate release. These studies demonstrated that astrocytic glutamate release was sensitive to exocytosis blockers or the expression of the essential machineries for regulating Ca^2+^-dependent exocytosis [33]. In addition to the vesicular mechanism, there is multiple lines of evidence suggesting that astrocytes express a Ca^2+^-dependent but non-vesicular glutamate release machinery [16]. Despite its unclear molecular identity, it has been reported that an increase in Ca^2+^ by GPCR activation induces glutamate release from astrocytes through osmolyte-permeable anion channels [10,28], which are activated by intracellular Ca^2+^ or increase in cell volume [16]. In our previous reports, we observed that Best1-mediated Ca^2+^-activated anion current could be induced by a host of GPCR agonists such as mGluR1/5, P2Y, B2, S1P, and LPAR in astrocytes [25], and that activation of those receptors elicits Ca^2+^-dependent glutamate release [7]. Finally, Best1 was demonstrated to be permeable to GABA and mediate tonic GABA release in cerebellar glial cells [26]. Recent study showed that GABA is abundant in cerebellar glial cells to be released tonically via Best1 to cause tonic inhibition [34]. However, in hippocampus, GABA is not present in glial cells; rather, Best1 might release glutamate near synapse. Therefore, it is plausible that Best1, at least in part, could be the downstream target of various G_q_-coupled GPCRs that signal through intracellular Ca^2+^ to release glutamate in hippocampal astrocytes.

In addition to Best1 channel, other anion channels might participate in anion channel-mediated glutamate release as previously suggested, such as Ca^2+^-induced but volume regulated osmolyte-permeable channels [16]. In fact, there are several studies showing that bestrophin channel could be opened by both cytosolic Ca^2+^ and cell volume increase [17,35], raising a possibility that Best1-mediated glutamate release is also triggered by Ca^2+^-dependent cell swelling and subsequent activation of VRAC. However, in our previous study we demonstrated that PAR-1 activation does not induce significant swelling [36] indicating that Ca^2+^ directly initiates Best1 channel-mediated glutamate release from astrocytes. The PAR1-induced astrocytic glutamate release was not completely inhibited by Best1 knock-down (Figure 4). Still, vesicular release mechanism was not involved because Best1 silencing did not affect the expression level of known genes involved in vesicular release (Figure 5A) and glutamate release from astrocytes was not reduced by treatment with Conconomycin A or Tetanus toxin (Figure 5B). There is a possibility that other ion channels that are independent of Best1 channel might participate in PAR1-induced glutamate release from astrocytes.

We utilized the native GPCR, PAR1, which has been extensively used in numerous studies to selectively activate astrocytes. Even though PAR1 is expressed in a subset of dentate granule cells [37], PAR1 has been shown to be expressed exclusively in astrocytes in human and rodent CA1 hippocampus [7,38,39], as well as in the nucleus of solitary tract [40] to mediate neuron-glia interaction. Therefore, the source of PAR1-induced glutamate is most likely astrocyte, as a direct consequence of increase [Ca^2+^_i_. Although there is no direct evidence of how PAR1 is activated in the physiological situation up to now, the recent study demonstrated that tPA-plasmin pathway is an endogenous PAR1 agonist [41], suggesting that physiological PAR1 activation is initiated by the activation of tPA-plasmin pathway in physiological condition such as synaptic plasticity [42,43].

In summary, we reveal a novel anion channel-mediated glutamate release mechanism in cultured astrocytes. The ideas and tools developed in this study should prove to be helpful in understanding the physiological role of glutamate release mechanism and its functional significances.

## Methods

### Primary astrocyte culture

Cell culture of mouse astrocytes was performed as previously described [7]. The cerebral cortex from P0 ~ P3 postnatal mice was dissected free of adherent meninges, minced and dissociated into single cell suspension by trituration. Dissociated cells were plated onto 12 mm glass coverslips coated with 0.1 mg/ml poly D-lysine. Cells were grown in DMEM supplemented with 25 mM glucose, 10% heat-inactivated horse serum, 10% heat-inactivated fetal bovine serum, 2 mM glutamine, and 1000 units ml-1 penicillin-streptomycin. Cultures were maintained at 37°C in humidified 5% CO_2_-containing atmosphere.

### Glutamate FRET imaging

In FRET-based glutamate imaging experiments, pDisplay- GluSnFR vector [30] was electroporated alone or with pSicoR-scrambled-shRNA or pSicoR-Best1-shRNA vector into cultured astrocytes (MicroPorator; Digital Bio, Korea). After 48 ~ 72 hour expression, FRET imaging was performed under a microscope (BX50WI; Olympus) equipped with xenon lamp with 436/20 excitation filter (D436/20x filter; Chroma). The emission beam was split with a DualView (Optical Insights) with a CFP/YFP filter set (OI-05-EX), recorded by EM-CCD camera (ANDOR IXON). Imaging Workbench software (INDEC BioSystems) was used for image acquisition and offline image analysis. TFLLR puff (TFLLR-NH_2_; Peptron, Korea; 500 μM) was made by using picospritzer-assisted positive pressure (~100 ms). The amount of released extracellular glutamate was described as ratio between the emission intensity of CFP and YFP (CFP/YFP), which was divided by baseline CFP/YFP ratio (relative CFP/YFP ratio).

### Radioactivity glutamate release assay

*In vitro* glutamate assay using ^3^H-labelled glutamate was performed as described previously [7]. Astrocytes were loaded with 0.5 μm l-^3^H]glutamate for 60 min by adding 1 μm of 1 mCi ml^−1^ l-^3^H]glutamate stock solution to 2 ml of culture medium. The cultures were preincubated for 30 min with 1 mm amino-oxyacetic acid and 0.5 mm methionine sulfoximine before adding ^3^H]glutamate, and during the loading to inhibit the metabolism of glutamate to glutamine and other metabolites [44]. Cells were washed with external solution 3 times. In some experiments, the external solution was supplemented with 50 μm l-transpyrrolidine-2,4-dicarboxylic acid (*trans*-PDC) to block glutamate transporter, a maximally effective concentration (6× IC_50_ of 4–8 μm; [45]) that is well below that suggested to stimulate heteroexchange (0.2 mm; [46,47]). Agonists were added to external solution for 6 min and the experiment was terminated by collection of the solution. Each experimental run included the control condition in which no agonist was added. Six replicates were obtained for each drug condition. For analysis, the average radioactivity count was obtained from six replicates for each condition and compared to the average of control.

### HPLC analysis for glutamate release

The Cortical primary astrocytes were cultured in 60 mm dishes for HPLC analysis. TFLLR (30 μM) was used to induce glutamate. Prior to TFLLR treatment, astrocytes were washed with PBS three times. For gene silencing experiment, Lentivirus containing Scrambled or Best1-shRNA was treated to cells and incubated for 48 hrs. The amino acid content was derivatized with *o*-phthaldialdehyde (OPA) and detected using UV (DAD) detection [48]. The OPA derivatized samples by programmed autosampler were injected on Zorbax Eclipse Plus C_18_ column with detection at 338 nm (a reference = 390 nm). Mobile phase A was 40 mM Na_2_HPO_4_ pH 7.8, and B was acetonitrile/methanol/water (45:45:10, v/v/v). The flow rate was 2 mL/min with a gradient condition that allowed for 1.9 min at 0% B and raised to 26% B at a 12.5 min-step. Then washing at 100% B and equilibration at 0% B was performed in a total retention time of 15 min. Reagents of OPA derivatization and all equipments for HPLC analysis were obtained from Agilent Technologies.

### Lentivirus with Best1-shRNA and Best1 mutagenesis

Best1-shRNA and lentivirus production were performed as previously described ([49], Virus facility, KIST). Best1 shRNA-insensitive pore mutant (Best1-shRNA insens W93C) was generated by using PCR-based site-directed mutagenesis kit (Stratagene, Cedar Creek, TX, USA).

### Electrophysiology

The extracellular recording solution for perforated patch clamp recording was comprised of (in mM) 150 NaCl, 10 HEPES, 3 KCl, 2 CaCl_2_, 2 MgCl_2_, 5.5 glucose, at pH 7.3. The internal solution contained 25 μg/ml gramicidin D and (in mM) 75 Cs_2_SO_4_, 10 NaCl, 0.1 CaCl_2_, and 10 HEPES, at pH 7.1. Pipette resistances ranged from 5 to 8 MΩ. It took 20 to 30 min to achieve acceptable perforation, with final series resistances ranging from 15 to 40 MΩ. Current voltage curves were established by applying 100- , 200-, or 1000 ms duration voltage ramps from –100 to +100 mV. Data were acquired by an Axopatch 200A amplifier controlled by Clampex 9.2 via Digidata 1322A data acquisition system (Axon Instruments, Union City, CA, USA). Experiments were conducted at room temperature (20 ~ 24°C).

### Reverse transcription-PCR

Total RNA was prepared from cultured astrocyte from postnatal day 0–3 mice using Trizol reagent (Invitrogen). cDNA was synthesized using Super Script III reverse transcriptase (Invitrogen). The reverse transcription (RT)-PCR primers used to check expression of Best1, Syt4 (Synaptotagmin4), Munc18-1, Vamp2 (Vesicle-associated membrane protein 2) GAPDH and glial fibrillary acidic protein (GFAP) were as followings

; Best1 forward, 5’-AGGACGATGATGATTTTGAG- 3’

; Best1 reverse, 5’-CTTTCTGGTTTTTCTGGTTG- 3’

; Syt4 forward, 5’-AGGCCAATTCCCCTGAGAGC- 3’

; Syt4 reverse, 5’-ACCGCTTCCTTCTGCTGTGG- 3’

; Munc18-1 forward, 5’-ACTCCGCTGACTCTTTCCAA- 3’

; Munc18-1 reverse, 5’-GTCGGCTTTATAGGCATCCA- 3’

; Vamp2 forward, 5’-GTCACTGCCTCTGCCAAGTC- 3’

; Vamp2 reverse, 5’-GGCAGACTCCTCAGGGATTT- 3’

; GAPDH forward, 5’-AGCTGAACGGGAAGCTCACT- 3’

; GAPDH reverse, 5’-TGCTGTAGCCAAATTCGTTG- 3’

; GFAP forward, 5’-AGAACAACCTGGCTGCGTAT-3’

; GFAP reverse, 5’-CGGCGATAGTCGTTAGCTTC-3’

Primers for Syt4, Munc18-1 and Vamp2 were designed as previous reports [50,51]. All reactions were performed in a programmable thermocycler (Eppendorf).

## Competing interests

The authors declare that they have no competing interests.

## Authors’ contributions

SJO performed patch clamp recording and wrote the manuscript. HP and KSH carried out FRET imaging experiments. DW and HYK performed HPLC analysis. SFT designed the radioactivity glutamate release assay experiment. CJL carried out radioactivity glutamate release assay and wrote the manuscript, and supervised entire project. All authors read and approved the final manuscript.

## References

[B1] Wang X Lou N Xu Q Tian GF Peng WG Han X Kang J Takano T Nedergaard M Astrocytic Ca2+ signaling evoked by sensory stimulation in vivo Nat Neurosci 2006 9 6 816 823 10.1038/nn1703 16699507

[B2] Haydon PG Carmignoto G Astrocyte control of synaptic transmission and neurovascular coupling Physiol Rev 2006 86 3 1009 1031 10.1152/physrev.00049.2005 16816144

[B3] Halassa MM Fellin T Haydon PG The tripartite synapse: roles for gliotransmission in health and disease Trends Mol Med 2007 13 2 54 63 10.1016/j.molmed.2006.12.005 17207662

[B4] Pascual O Casper KB Kubera C Zhang J Revilla-Sanchez R Sul JY Takano H Moss SJ McCarthy K Haydon PG Astrocytic purinergic signaling coordinates synaptic networks Science 2005 310 5745 113 116 10.1126/science.1116916 16210541

[B5] Volterra A Meldolesi J Astrocytes, from brain glue to communication elements: the revolution continues Nat Rev Neurosci 2005 6 8 626 640 10.1038/nrn1722 16025096

[B6] Perea G Araque A Astrocytes potentiate transmitter release at single hippocampal synapses Science 2007 317 5841 1083 1086 10.1126/science.1144640 17717185

[B7] Lee CJ Mannaioni G Yuan H Woo DH Gingrich MB Traynelis SF Astrocytic control of synaptic NMDA receptors J Physiol 2007 581 Pt 3 1057 1081 17412766 10.1113/jphysiol.2007.130377 PMC2170820

[B8] Panatier A Theodosis DT Mothet JP Touquet B Pollegioni L Poulain DA Oliet SH Glia-derived D-serine controls NMDA receptor activity and synaptic memory Cell 2006 125 4 775 784 10.1016/j.cell.2006.02.051 16713567

[B9] Parpura V Basarsky TA Liu F Jeftinija K Jeftinija S Haydon PG Glutamate-mediated astrocyte-neuron signalling Nature 1994 369 6483 744 747 10.1038/369744a0 7911978

[B10] Nedergaard M Takano T Hansen AJ Beyond the role of glutamate as a neurotransmitter Nat Rev Neurosci 2002 3 9 748 755 10.1038/nrn916 12209123

[B11] Montana V Malarkey EB Verderio C Matteoli M Parpura V Vesicular transmitter release from astrocytes Glia 2006 54 7 700 715 10.1002/glia.20367 17006898

[B12] Hamilton NB Attwell D Do astrocytes really exocytose neurotransmitters? Nat Rev Neurosci 2010 11 4 227 238 10.1038/nrn2803 20300101

[B13] Jourdain P Bergersen LH Bhaukaurally K Bezzi P Santello M Domercq M Matute C Tonello F Gundersen V Volterra A Glutamate exocytosis from astrocytes controls synaptic strength Nat Neurosci 2007 10 3 331 339 10.1038/nn1849 17310248

[B14] Kimelberg HK Macvicar BA Sontheimer H Anion channels in astrocytes: biophysics, pharmacology, and function Glia 2006 54 7 747 757 10.1002/glia.20423 17006903 PMC2556042

[B15] Walz W Chloride/anion channels in glial cell membranes Glia 2002 40 1 1 10 10.1002/glia.10125 12237839

[B16] Takano T Kang J Jaiswal JK Simon SM Lin JH Yu Y Li Y Yang J Dienel G Zielke HR Receptor-mediated glutamate release from volume sensitive channels in astrocytes Proc Natl Acad Sci U S A 2005 102 45 16466 16471 10.1073/pnas.0506382102 16254051 PMC1283436

[B17] Hartzell HC Qu Z Yu K Xiao Q Chien LT Molecular physiology of bestrophins: multifunctional membrane proteins linked to best disease and other retinopathies Physiol Rev 2008 88 2 639 672 10.1152/physrev.00022.2007 18391176

[B18] Eggermont J Calcium-activated chloride channels: (un)known, (un)loved? Proc Am Thorac Soc 2004 1 1 22 27 10.1513/pats.2306010 16113407

[B19] Kunzelmann K Milenkovic VM Spitzner M Soria RB Schreiber R Calcium-dependent chloride conductance in epithelia: is there a contribution by Bestrophin? Pflugers Arch 2007 454 6 879 889 10.1007/s00424-007-0245-z 17361457

[B20] Qu Z Fischmeister R Hartzell C Mouse bestrophin-2 is a bona fide Cl(-) channel: identification of a residue important in anion binding and conduction J Gen Physiol 2004 123 4 327 340 10.1085/jgp.200409031 15051805 PMC2217464

[B21] Sun H Tsunenari T Yau KW Nathans J The vitelliform macular dystrophy protein defines a new family of chloride channels Proc Natl Acad Sci U S A 2002 99 6 4008 4013 10.1073/pnas.052692999 11904445 PMC122639

[B22] Pifferi S Pascarella G Boccaccio A Mazzatenta A Gustincich S Menini A Zucchelli S Bestrophin-2 is a candidate calcium-activated chloride channel involved in olfactory transduction Proc Natl Acad Sci U S A 2006 103 34 12929 12934 10.1073/pnas.0604505103 16912113 PMC1568948

[B23] Marmorstein AD Marmorstein LY Rayborn M Wang X Hollyfield JG Petrukhin K Bestrophin, the product of the Best vitelliform macular dystrophy gene (VMD2), localizes to the basolateral plasma membrane of the retinal pigment epithelium Proc Natl Acad Sci U S A 2000 97 23 12758 12763 10.1073/pnas.220402097 11050159 PMC18837

[B24] Chien LT Zhang ZR Hartzell HC Single Cl- channels activated by Ca2+ in Drosophila S2 cells are mediated by bestrophins J Gen Physiol 2006 128 3 247 259 10.1085/jgp.200609581 16940553 PMC2151570

[B25] Park H Oh SJ Han KS Woo DH Mannaioni G Traynelis SF Lee CJ Bestrophin-1 encodes for the Ca2 + -activated anion channel in hippocampal astrocytes J Neurosci 2009 29 41 13063 13073 10.1523/JNEUROSCI.3193-09.2009 19828819 PMC2825675

[B26] Lee S Yoon BE Berglund K Oh SJ Park H Shin HS Augustine GJ Lee CJ Channel-mediated tonic GABA release from glia Science 2010 330 6005 790 796 10.1126/science.1184334 20929730

[B27] Hollenberg MD Saifeddine M al-Ani B Kawabata A Proteinase-activated receptors: structural requirements for activity, receptor cross-reactivity, and receptor selectivity of receptor-activating peptides Can J Physiol Pharmacol 1997 75 7 832 841 10.1139/y97-110 9315351

[B28] Ramos-Mandujano G Vazquez-Juarez E Hernandez-Benitez R Pasantes-Morales H Thrombin potently enhances swelling-sensitive glutamate efflux from cultured astrocytes Glia 2007 55 9 917 925 10.1002/glia.20513 17437307

[B29] Zhang Y Stanton JB Wu J Yu K Hartzell HC Peachey NS Marmorstein LY Marmorstein AD Suppression of Ca2+ signaling in a mouse model of Best disease Hum Mol Genet 2010 19 6 1108 1118 10.1093/hmg/ddp583 20053664 PMC2830833

[B30] Hires SA Zhu Y Tsien RY Optical measurement of synaptic glutamate spillover and reuptake by linker optimized glutamate-sensitive fluorescent reporters Proc Natl Acad Sci U S A 2008 105 11 4411 4416 10.1073/pnas.0712008105 18332427 PMC2393813

[B31] Mothet JP Pollegioni L Ouanounou G Martineau M Fossier P Baux G Glutamate receptor activation triggers a calcium-dependent and SNARE protein-dependent release of the gliotransmitter D-serine Proc Natl Acad Sci U S A 2005 102 15 5606 5611 10.1073/pnas.0408483102 15800046 PMC556243

[B32] Araque A Parpura V Sanzgiri RP Haydon PG Glutamate-dependent astrocyte modulation of synaptic transmission between cultured hippocampal neurons Eur J Neurosci 1998 10 6 2129 2142 10.1046/j.1460-9568.1998.00221.x 9753099

[B33] Malarkey EB Parpura V Mechanisms of glutamate release from astrocytes Neurochem Int 2008 52 1–2 142 154 17669556 10.1016/j.neuint.2007.06.005 PMC2267911

[B34] Yoon BE Woo J Justin Lee C Astrocytes as GABA-ergic and GABA-ceptive Cells Neurochem Res 2012 [Epub ahead of print] 10.1007/s11064-012-0808-z 22700085

[B35] Chien LT Hartzell HC Drosophila bestrophin-1 chloride current is dually regulated by calcium and cell volume J Gen Physiol 2007 130 5 513 524 10.1085/jgp.200709795 17968025 PMC2151665

[B36] Gingrich MB Junge CE Lyuboslavsky P Traynelis SF Potentiation of NMDA receptor function by the serine protease thrombin J Neurosci 2000 20 12 4582 4595 10844028 10.1523/JNEUROSCI.20-12-04582.2000 PMC6772448

[B37] Han KS Mannaioni G Hamill CE Lee J Junge CE Lee CJ Traynelis SF Activation of protease activated receptor 1 increases the excitability of the dentate granule neurons of hippocampus Mol Brain 2011 4 32 10.1186/1756-6606-4-32 21827709 PMC3170262

[B38] Junge CE Lee CJ Hubbard KB Zhang Z Olson JJ Hepler JR Brat DJ Traynelis SF Protease-activated receptor-1 in human brain: localization and functional expression in astrocytes Exp Neurol 2004 188 1 94 103 10.1016/j.expneurol.2004.02.018 15191806

[B39] Shigetomi E Bowser DN Sofroniew MV Khakh BS Two forms of astrocyte calcium excitability have distinct effects on NMDA receptor-mediated slow inward currents in pyramidal neurons J Neurosci 2008 28 26 6659 6663 10.1523/JNEUROSCI.1717-08.2008 18579739 PMC2866443

[B40] Hermann GE Van Meter MJ Rood JC Rogers RC Proteinase-activated receptors in the nucleus of the solitary tract: evidence for glial-neural interactions in autonomic control of the stomach J Neurosci 2009 29 29 9292 9300 10.1523/JNEUROSCI.6063-08.2009 19625519 PMC2773000

[B41] Mannaioni G Orr AG Hamill CE Yuan H Pedone KH McCoy KL Berlinguer Palmini R Junge CE Lee CJ Yepes M Plasmin potentiates synaptic N-methyl-D-aspartate receptor function in hippocampal neurons through activation of protease-activated receptor-1 J Biol Chem 2008 283 29 20600 20611 10.1074/jbc.M803015200 18474593 PMC2459301

[B42] Tomimatsu Y Idemoto S Moriguchi S Watanabe S Nakanishi H Proteases involved in long-term potentiation Life Sci 2002 72 4–5 355 361 12467876 10.1016/s0024-3205(02)02285-3

[B43] Pang PT Lu B Regulation of late-phase LTP and long-term memory in normal and aging hippocampus: role of secreted proteins tPA and BDNF Ageing Res Rev 2004 3 4 407 430 10.1016/j.arr.2004.07.002 15541709

[B44] Farinelli SE Nicklas WJ Glutamate metabolism in rat cortical astrocyte cultures J Neurochem 1992 58 5 1905 1915 10.1111/j.1471-4159.1992.tb10068.x 1348525

[B45] Mitrovic AD Johnston GA Regional differences in the inhibition of L-glutamate and L-aspartate sodium-dependent high affinity uptake systems in rat CNS synaptosomes by L-trans-pyrrolidine-2,4-dicarboxylate, threo-3-hydroxy-D-aspartate and D-aspartate Neurochem Int 1994 24 6 583 588 10.1016/0197-0186(94)90011-6 7981641

[B46] Volterra A Bezzi P Rizzini BL Trotti D Ullensvang K Danbolt NC Racagni G The competitive transport inhibitor L-trans-pyrrolidine-2, 4-dicarboxylate triggers excitotoxicity in rat cortical neuron-astrocyte co-cultures via glutamate release rather than uptake inhibition Eur J Neurosci 1996 8 9 2019 2028 10.1111/j.1460-9568.1996.tb01345.x 8921292

[B47] Bezzi P Carmignoto G Pasti L Vesce S Rossi D Rizzini BL Pozzan T Volterra A Prostaglandins stimulate calcium-dependent glutamate release in astrocytes Nature 1998 391 6664 281 285 10.1038/34651 9440691

[B48] Bartolomeo MP Maisano F Validation of a reversed-phase HPLC method for quantitative amino acid analysis J Biomol Tech 2006 17 2 131 137 16741240 PMC2291777

[B49] Park H Oh SJ Han KS Woo DH Park H Mannaioni G Traynelis SF Lee CJ Bestrophin-1 encodes for the Ca2 + -activated anion channel in hippocampal astrocytes J Neurosci 2009 29 41 13063 13073 10.1523/JNEUROSCI.3193-09.2009 19828819 PMC2825675

[B50] Zhang Q Pangrsic T Kreft M Krzan M Li N Sul JY Halassa M Van Bockstaele E Zorec R Haydon PG Fusion-related release of glutamate from astrocytes J Biol Chem 2004 279 13 12724 12733 14722063 10.1074/jbc.M312845200

[B51] Zhang Q Fukuda M Van Bockstaele E Pascual O Haydon PG Synaptotagmin IV regulates glial glutamate release Proc Natl Acad Sci U S A 2004 101 25 9441 9446 10.1073/pnas.0401960101 15197251 PMC438995

